# Neurological manifestations in people with HIV in HAART era: a cross-sectional multicenter study

**DOI:** 10.3389/fneur.2026.1772052

**Published:** 2026-05-01

**Authors:** Lu Lu, Jian Hu, Peiyu Wang, Longting Du, Jing Xiao, Guoxiang Lu, Kui Zhou, Peng Chao, PoWuJu Wu, Zhiyong Zong, Liyu Chen, Lei Chen, Lin Chen, Dong Zhou, Weixi Xiong

**Affiliations:** 1 Department of Neurology, West China Hospital, Institute of Brain Science and Brain-Inspired Technology, Sichuan University, Chengdu, Sichuan, China; 2 Department of Neurology, The Second People’s Hospital of Liangshan Autonomous Prefecture, Xichang, Sichuan, China; 3 Department of Neurology, Dechang People’s Hospital, Dechang, Sichuan, China; 4 Department of Neurology, Chengdu Shangjin Nanfu Hospital, Chengdu, Sichuan, China; 5 Center for Infectious Diseases, West China Hospital of Sichuan University, Chengdu, China

**Keywords:** AIDS, Anti-retroviral therapy, Dementia, Intracranial infection, Stroke

## Abstract

**Background:**

The landscape of neurological manifestations of HIV remains unknown after HAART is applied. This study aimed to characterize the spectrum of neurological disorders in people with HIV.

**Methods:**

This cross-sectional study was conducted in four tertiary hospitals in China, between January 2018, and June 2025. We recruited individuals aged >16 years with HIV who presented with neurological symptoms or had related complaints. The diagnostic criteria for HIV and its stage classification were based on the 2024 Chinese HIV Prevention and Treatment Guidelines as Stage I (Early infection Stage), II (Asymptomatic Stage), and III (AIDS Stage). Data were extracted using standardized forms. Statistical analyses included ANOVA and Chi-square tests, and Fisher’s exact test.

**Results:**

Three hundred and forty-seven individuals were enrolled, of whom 69% were newly diagnosed with HIV. The most common neurological complaints were headache (30%), dizziness (13%), and neurobehavioral manifestations (8.0%). The most frequent discharge diagnoses were intracranial infections (21%), followed by cerebrovascular disease (16%) and primary headache (11%). Virus (25%), Tuberculosis (20%) and *Treponema pallidum* (10%) were the three most common types of infections. Few differences were observed among the three HIV stages, except for intracranial infection (*p* = 8.09e-06), intracranial space-occupying lesions of unknown cause (*p* = 0.03), and anxiety (*p* = 0.01). At discharge, cognitive decline was observed in 15%, and dementia was diagnosed in an additional 2%.

**Conclusion:**

The study emphasizes the importance of HIV testing in the neurological setting. Neurological manifestations could be the first manifestations in people with HIV in HAART era. Intracranial infections were the most common diagnosis.

## Graphical abstract

**Figure:**
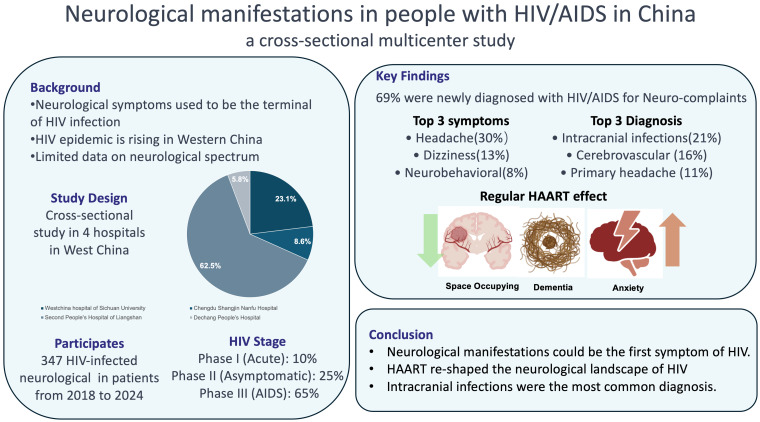

## Introduction

Human immunodeficiency virus (HIV) attacks the body’s immune system, leading to various clinical manifestations. Neurological symptoms used to be considered the terminal symptoms during HIV infection (1). As a result of standard treatment consisting of a combination of drugs (often called “highly active antiretroviral therapy” or HAART), HIV has become more of a chronic disease with a near-normal life expectancy. However, HIV-associated neurological disorders are still common, and in some previous reports, neurological disease was the first symptom of HIV infection in up to a fifth of individuals (2).

The incidence of neurological complications varied geographically, influenced by the timely diagnosis and early introduction of HAART (3–5). In China, HAART has been provided free of charge since 2002 by the National Free Antiretroviral Treatment Program (NFATP) (6). Despite it, both HIV/AIDS prevalence and new infection rates continued to rise in China from 1990 to 2016 (7, 8). At the end of 2020, 1.05 million people were living with HIV, and there were 351,000 cumulative reported deaths in China (9). A nested case–control study from Taiwan estimated a stable incidence of neurological disorders of 13.67 per 1,000 person-years, despite the use of early treatment and more tolerable modern anti-retroviral therapy (10). In another cohort study from Beijing, up to a tenth of people with HIV (PWH) were hospitalized for neurological complications (11). Locally, a significantly lower rate of HIV/AIDS in the central and eastern regions of China compared to its western regions has also been reported (9). The primary landscape of neurological manifestations in PWH admitted to hospitals in China is still largely unknown.

This cross-sectional study aimed to establish the spectrum of neurological manifestations in people with HIV/AIDS admitted to four participating centers in China.

## Methods

### Ethics approval and consent

The West China Hospital Ethics Committee [reference number: 2023 (12)] approved the project. Participants provided written informed consent for study and publication or assent when applicable.

### Study population

This cross-sectional study was conducted across four tertiary hospitals in Sichuan Province: West China Hospital of Sichuan University, Chengdu Shangjin Nanfu Hospital, Liangshan Second People’s Hospital, and Dechang People’s Hospital. Individuals aged >16 years with HIV who presented with neurological symptoms or had neurological complaints as the primary diagnosis at discharge were consecutively and retrospectively enrolled upon discharge from the participating hospitals between January 1, 2018, and June 1, 2025. All neurological diagnoses were confirmed by neurologists at each center. Data were extracted by trained neurologists using a designed case report form.

The diagnostic and staging criteria for HIV were adapted from the 2024 edition of the Chinese HIV Prevention and Treatment Guidelines, issued by the Chinese Centers for Disease Control and the HIV Task Force under the Infectious Diseases Branch of the Chinese Medical Association (13). Those who tested positive in an antibody screening test and negative in complementary tests were excluded. Participants were classified into Stage I (early or acute infection), Stage II (asymptomatic stage), and Stage III (AIDS stage). People with central nervous system (CNS)-occupying lesions, toxoplasmosis encephalopathy, or primary CNS lymphoma were categorized under the appropriate AIDS Stage.

Neurological diagnosis was made by attending neurologists in the respective hospitals according to international and Chinese national guidelines. The detailed diagnostic criteria are provided in Supplementary Table S1.

### Data acquisition

A standardized form was designed to extract demographic data, clinical features, neurological examination, HIV-related history, HIV laboratory metrics, and results from imaging and electroencephalograms. Trained physicians completed the forms.

The reasons for admission were stratified into new-onset neurological manifestations and worsening pre-existing neurological manifestations. Neurologists evaluated the outcomes at discharge based on the modified Rankin scale (mRS).

Cognitive function evaluations were based on documented neurological records, with recorded scores from the Mini-Mental State Examination (MMSE), Montreal Cognitive Assessment (MoCA), Clinical Dementia Rating (CDR), or the International HIV Dementia Scale, if available.

### Statistical analysis

ANOVA and Chi-square tests were utilized for variable analysis, with Fisher’s exact test applied to categorical data with sparse distributions. Statistical analysis was performed using IBM SPSS Statistics, and a two-sided *p*-value < 0.05 was considered statistically significant.

## Results

Of the 368 candidates screened, two tested negative on HIV confirmatory testing and seven were excluded due to missing confirmatory test data. An additional 12 individuals refused to participate. 347 individuals were eventually enrolled: eighty at West China Hospital, 30 from Chengdu Shangjin Nanfu Hospital, 200 and 17 from Liangshan Second People’s Hospital, and 20 from Dechang People’s Hospital.

The demographic characteristics across different HIV infection Stages are summarised in Table 1. Ten participants died during admission; among them, three were in the acute infection stage. The primary death causes included hemorrhage in the basal ganglia and brain herniation following the intracranial infection. Seven deaths were observed in the AIDS Stage, associated with showing more disseminated widespread infections and cardiopulmonary functional failure in the heart and lungs. Multiple organ dysfunction syndrome (MODS) was diagnosed in four out of seven. Three of them were receiving first-line HAART treatment (TDF + 3TC + EFV). The other three died of their multiple intracranial space-occupying lesions.

**Table 1:** Demographic features among different infection stage of HIV in the study cohort.

Features		Stage I	Stage II	AIDS	Total	*p*
Number		86	110	151	347	NA
Gender	Male	62 (72.1)	80 (72.7)	114 (75.5)	256 (73.8)	0.81
	Female	24 (27.9)	30 (27.3)	37 (24.5)	91 (26.2)	
Age (yr)		41 (33.0,54.8)	42 (35,49.0)	49 (36,63)	45 (34.25,56)	0.001
Admission	Outpatient	56 (65.1)	87 (79.1)	111 (73.5)	254 (73.2)	0.09
	Emergency	30 (34.9)	23 (20.9)	40 (26.5)	93 (85.0)	
Length of Stay		6.0 (3,9)	6.0 (3,9)	6.5 (4,10)	6 (3,9)	0.009
Reason for Admission	New merged disorders	71 (82.6)	98 (89.1)	126 (83.4)	295 (85.0)	0.344
	Progression of prior disease	15 (17.4)	12 (10.9)	25 (16.6)	52 (15.0)	
Death		3 (3.5)	0	7 (4.6)	10 (2.9)	0.047
MRS at discharge	MRS<4	76 (88.4)	103 (93.6)	125 (82.8)	304 (87.6)	0.03
	MRS ≥ 4	10 (11.6)	7 (16.4)	26 (17.2)	43 (12.4)	
Newly diagnosed HIV		61 (70.9)	78 (70.9)	101 (66.9)	240 (69.2)	0.7
Regular HAART treatment		15 (17.4)	21 (19.1)	41 (27.2)	77 (22.2)	0.14

MRS, Modified rankin scale; HAART, highly active antiretroviral therapy.

The most common neurological manifestations across all groups, particularly in Stage I, included headache, dizziness, and neurobehavioral manifestations. Forty-one patients presented with limb weakness, paresthesia, or pain; of these, eight were on regular HAART at symptom onset, with two cases attributed to HAART-related peripheral neuropathy. No other HAART-associated neurological symptoms were identified. The distribution of main manifestations and primary discharge diagnoses of all stages is detailed in Table 2.

**Table 2:** The Chief complaints in PWH in different HIV stage.

Characteristic		Stage I (*N* = 86)	Stage II (*N* = 110)	AIDS (*N* = 151)	Total (*N* = 347)	*p*
Course	Acute	41 (47.7)	40 (36.4)	60 (39.7)	141 (40.6)	0.27
	Subacute	25 (29.1)	27 (24.5)	43 (28.5)	95 (27.4)	0.72
	Chronic	20 (23.3)	43 (39.1)	48 (31.8)	111 (32.0)	0.06
Chief Complaints	Headache	29 (33.7)	40 (36.4)	34 (22.5)	103 (29.7)	0.03
	Dizziness	6 (7.0)	17 (15.5)	22 (14.6)	45 (13.0)	0.16
	Psycho-behavioral abnormalities	16 (18.6)	2 (1.8)	9 (6.0)	27 (7.8)	4.131e-05
	Limb weakness	5 (5.8)	5 (4.5)	14 (9.3)	24 (6.9)	0.30
	Motor dysfunction in limb	5 (5.8)	6 (5.5)	9 (6.0)	20 (5.8)	0.99
	Cognitive impairment	2 (2.3)	5 (4.5)	8 (5.3)	15 (4.3)	0.55
	Loss of Consciousness	1 (1.2)	5 (4.5)	9 (6.0)	15 (4.3)	0.22
	Speech disorder	1 (1.2)	5 (4.5)	8 (5.3)	14 (4.0)	0.28
	Visual impairment	3 (3.5)	4 (3.6)	6 (4.0)	13 (3.7)	0.98
	Seizure	4 (4.7)	1 (0.9)	7 (4.6)	12 (3.5)	NA
	Facial pain	1 (1.2)	3 (2.7)	5 (3.3)	9 (2.6)	NA
	Trunk Pain	2 (2.3)	2 (1.8)	5 (3.3)	9 (2.6)	NA
	Limb paresthesia	1 (1.2)	4 (3.6)	4 (2.6)	9 (2.6)	NA
	Limb pain	4 (4.7)	2 (1.8)	2 (1.3)	8 (2.3)	NA
	Gait disturbance	2 (2.3)	3 (2.7)	0	5 (1.4)	NA
	Tremor	1 (1.2)	1 (0.9)	3 (2.0)	5 (1.4)	NA
	Others	3 (3.5)	5 (4.5)	6 (4.0)	14 (4.0)	0.93

NA, not applicable.

Seventy-three participants (30.8%) presented with intracranial infections. Etiologies are listed in Table 3, with the most prevalent viral infection attributed to Herpes Simplex Virus, alongside two cases of John Cunningham virus and classic progressive multifocal leukoencephalopathy.

**Table 3:** The discharge diagnoses in PWH in different HIV stage.

Discharge diagnoses	Stage I (*N* = 86)	Stage II (*N* = 110)	AIDS (*N* = 151)	Total (*N* = 347)	*p*
Intracranial infection	31 (36.0)	9 (8.2)	33 (21.9)	73 (21.0)	8.09e-06
Viral	7 (22.6)	3 (33.3)	8 (24.2)	18 (24.7)	0.80
Tuberculosis	8 (25.8)	2 (22.2)	4 (12.1)	14 (19.2)	0.37
*Treponema pallidum*	2 (6.45)	3 (33.3)	2 (6.0)	7 (9.6)	NA
Bacterial	2 (6.45)	1 (11.1)	1 (3.0)	4 (5.5)	NA
Cryptococcal	0	0	4 (12.1)	4 (5.5)	NA
Toxoplasmosis	0	0	3 (9.1)	3 (4.1)	NA
Cysticercosis	0	0	1 (3.0)	1 (1.4)	NA
Echinococcosis	0	0	1 (3.0)	1 (1.4)	NA
Rickettsial	1 (3.2)	0	0	1 (1.4)	NA
Unspecified	11 (35.5)	0	9 (27.3)	20 (27.4)	0.01
Cerebrovascular disease	12 (14.0)	15 (13.6)	28 (18.5)	55 (15.9)	0.58
Primary headache	14 (16.3)	13 (11.8)	12 (7.9)	39 (11.2)	0.15
Intracranial space-occupying lesion of unknown reason	2 (2.3)	10 (9.1)	19 (12.6)	31 (8.9)	0.03
Postherpetic neuralgia	7 (8.1)	6 (5.5)	10 (6.6)	23 (6.6)	0.39
Anxiety	2 (2.3)	10 (9.1)	3 (2.0)	15 (4.3)	0.01
Neuroimmune-disorder	3 (3.5)	5 (4.5)	4 (2.6)	12 (3.5)	0.71
Dizziness/vertigo	2 (2.3)	1 (0.9)	9 (6.0)	12 (3.5)	0.07
Encephalopathy	2 (2.3)	4 (3.6)	5 (3.3)	11 (3.2)	0.87
Peripheral Neuropathy	1 (1.2)	7 (6.4)	4 (2.6)	12 (3.5)	0.07
Epilepsy	2 (2.3)	0	7 (4.6)	9 (2.6)	NA
Post traumatic brain injury	0	11 (10.0)	3 (2.0)	14 (4.0)	0
Dementia	2 (2.3)	4 (3.6)	1 (0.6)	7 (2.0)	NA
Intracranial hemorrhage	2 (2.3)	6 (5.5)	4 (2.6)	12 (3.5)	0.38
Parkinson disease	3 (3.5)	1 (0.9)	1 (0.6)	5 (1.4)	NA
Others	1 (1.2)	8 (7.3)	8 (5.3)	17 (4.9)	0.14

NA, not applicable.

Two hundred and forty-seven individuals (69.0%) were newly diagnosed with HIV, while 107 had a prior diagnosis. All were initially prescribed first-line HAART per the Chinese guidelines (TDF/3TC/EFV or BIC/FTC/TAF), with 77 (72.0%) continuing regular treatment at admission. The diagnosis distribution in the treated and untreated groups is shown in Table 4.

**Table 4:** The discharge diagnoses distribution between regular or non-regular HAART treated PWH.

Discharge diagnoses	Regular HAART treatment (*N* = 77)	Non-regular HAART treatment (*N* = 270)	Total	*p*
Intracranial infection	11 (14.3)	62 (17.9)	73	0.136
Cerebrovascular disease	11 (14.3)	44 (12.7)	55	0.803
Primary headache	9 (11.7)	30 (8.6)	39	0.513
Intracranial space-occupying lesion of unknown reason	2 (2.6)	29 (8.4)	31	0.047
Postherpetic neuralgia	3 (3.9)	20 (5.8)	23	0.405
Anxiety	7 (9.1)	8 (2.3)	15	0.028
Neuroimmune-disorder	5 (6.5)	7 (2.0)	12	0.148
Dizziness/Vertigo	6 (7.8)	6 (1.7)	12	0.029
Encephalopathy	3 (3.9)	8 (2.3)	11	0.713
Peripheral neuropathy	4 (5.2)	8 (2.3)	12	0.311
Epilepsy	1 (1.3)	8 (2.3)	9	NA
Post traumatic brain injury	2 (2.6)	12 (3.5)	14	0.743
Dementia	2 (2.6)	5 (1.4)	7	NA
Intracranial hemorrhage	4 (5.2)	8 (2.3)	12	0.311
Parkinson disease	3 (3.9)	2 (0.6)	5	NA
Others	4 (5.2)	13 (3.7)	17	0.544

HAART, highly active antiretroviral therapy; NA, not applicable.

## Discussion

In this cross-sectional study, we recruited a large cohort of PWH who were admitted primarily due to neurologic symptoms, which led to their initial HIV diagnosis in most cases. Most were first diagnosed with HIV during admission as part of their investigations. Intracranial infection was the most common first diagnosis, followed by cerebrovascular diseases, primary headaches and intracranial space-occupying lesions of unknown reasons. Subgroup analysis revealed significant differences in the prevalence of intracranial infections, space-occupying lesions of unknown etiology, and anxiety across different stages of HIV infection. However, no significant difference in most neurological diagnoses was observed between participants on regular HAART and those who were treatment-naïve, except for the aforementioned conditions.

Neurological manifestations of HIV have long been recognized in HIV infection (14). The early seeding of HIV in CNS, facilitated by initial blood–brain barrier (BBB) disruption, establishes a persistent viral reservoir within resident microglia and macrophages. Even with suppressive systemic HAART, these reservoirs and the ongoing production of neurotoxic viral proteins, fuel the chronic neuroinflammation state. These processes (15) directly cause synaptic dysfunction, neuronal injury. Importantly, this HIV-driven neuroinflammation shares mechanistic hallmarks with the aging brain’s inflammatory environment, creating a synergistic effect that accelerates damage (16). The prevalence of neuro-HIV affects up to 70% of PWH, and even more have evidence of neurological compromise at autopsy (4, 17). With the rapid development of effective treatment for HIV, CNS opportunistic infections were nearly eliminated, at least in developed areas. However, neuropsychiatric impairment and peripheral nerve damage were still common, which led to high morbidity and mortality of those neurological disorders in HIV, calling for more understanding of the pathogenesis, clinical features and treatment of neuro-HIV.

In our cohort, nearly over two-thirds of people were newly diagnosed with HIV due to emerging neurological compromise, and ten died during admission. The proportion of people complaining of neurological symptoms was higher than in another report from China. The death rate was, however, comparatively low (18). The reason could be the difference in the inclusion criteria, as we had more people in the early stage of HIV. Our study also validated the essential role of HIV testing in people with new neurological symptoms.

Intracranial infection was our most common neurological problem. It aligns with previous studies in China (11) and from other low-income countries (2, 19, 20). However, the spectrum differed; cryptococcal meningitis and cerebral toxoplasmosis were fewer than in previous studies. The difference could be that our cohort had more people in the early stage rather than late stage. Virus, tuberculosis, and *treponema pallidum* were our cohort’s three most common infections. This could be partially explained by the high incidence of tuberculosis in China and the immunosuppression associated with HAART. In other low-income countries, tuberculosis was rare in PWH (21). We had a certain number of cases with intracranial lesions of unknown causes so that the actual number of CNS infections could be higher than previously reported. CNS infections had a low chance of being detected by any methods, including polymerase chain reaction (qPCR), antigen test, and even metagenomic next-generation sequencing (NGS), left up to 5% of people undiagnosed (22).

Stroke was also common in the cohort, and HIV status has been proven to elevate slightly the risk of stroke (23, 24). The possible reasons include the high likelihood of diabetes, hypertension in PWH, accelerated atherosclerosis, HIV-induced cardiac disease and HIV-associated cerebral vasculopathy (25). In some reports, HIV status did not influence the carotid intima-media thickness in people with stroke, which is a validated surrogate marker of atherosclerosis and an accurate predictor of future cardiovascular events (26). We did not observe the same association in our patients with stroke, and future work should include investigations into the etiology, prevention, and treatment.

We reported that a certain proportion of people had main complaints of neuralgia, and most were diagnosed with postherpetic neuralgia. The risk of herpes zoster is substantially higher among PWH than in the general population (hazard ratio 16.9), particularly in those not receiving HAART. Additionally, approximately 90% of PWH are seropositive for varicella zoster virus (27, 28). The high proportion of treatment-naive PWH could explain the number of postherpetic neuralgia. We also reported a smaller portion of peripheral neuropathy, which was also common in all stages of HIV infection, and the insult of peripheral nerves was diverse in pathogenesis and clinical features. The possible mechanism included immunopathogenesis associated with neurotoxicity from the HIV and neurotoxicity of the ART treatment. At least six patterns of HIV/AIDS-associated PN were reported (12, 29, 30), and the most common type, distal symmetry polyneuropathy, could affect nearly half of the PWH (31). In a cohort on regular HAART treatment from Ghana, the prevalence was 10%, which was the fifth most common commodity, following respiratory tract infections, anaemia, hypertension and cerebrovascular disease (32).

Around 17% of our study population reported apparent cognitive impairment. The mental decline in PWH was common, and the most widely accepted term for this syndrome was HIV-Associated Neurocognitive Disorder (HAND) (33). This included three levels of cognitive decline: asymptomatic neurocognitive impairment (ANI), HIV-associated mild neurocognitive disorder (MND), and HIV-associated dementia (HAD). In the era of wide application of HAART, the overall severity of HAND decreased, but the proportion increased, with unclear pathogenesis (34). It was reported that in treated PWH, the prevalence of HAND ranges from 33%–55% (34–36), and the dementia comprising less than 10% (37). In a previous study in South China, 8.3% had similar figures were shown in West China, a treatment-naive population (38). However, the true incidence might be underestimated if further and detailed crowd neuropsychological tests were applied.

We recognized that our study has several limitations. Firstly, the major limitation was that due to the study design, epidemiological HIV data of the catchment area was not available This included the number of people HIV positive and the number of people on HAART, so our result may not be generalized to other populations, as we mainly included adults and 70% were treatment naive. Secondly, the retrospective nature of our study might cause bias, including an underestimate of some mild cases and not all participants underwent all complementary tests, for example, we did not report the CD4 count, but CD4 count variation may not have correlations with the outcome of neurological complications (2, 39). Thirdly, some participant centres had limited medical resources, leading to partial or error diagnoses. Lastly, consistent with China’s HIV epidemic demographics, the male predominance in our cohort may limit generalizability of the results to other population.

Despite all these limitations, our studies revealed the spectrum of neurological diseases in PWH in West China. These findings warrant a more in-depth analysis in the future.

## Conclusion

This study investigated neurological symptoms in a large cohort of PWH in West China, most of whom were newly diagnosed. Our findings highlight the importance of HIV testing for individuals with neurological complaints and underscore the need for further research on the neurological complications of HIV in this region.

## Data Availability

The dataset is not publicly available due to patient confidentiality and ethical restrictions involving HIV clinical information, but de-identified data may be provided to qualified researchers upon reasonable request to the corresponding author, subject to necessary approvals. Requests to access the datasets should be directed to 502216168@qq.com.
