# Supplementary material for: Neurological manifestations in people with HIV in HAART era: a cross-sectional multicenter study
Source: Front Neurol. 2026 May 1;17:1772052. doi: 10.3389/fneur.2026.1772052 (PMC13175781; doi:10.3389/fneur.2026.1772052)
Supplement: Supplementary file 1 [file Table_1.docx]

Supplementary Table 1. The diagnose criteria of neurological diagnosis

| Diagnose | Criteria |
| --- | --- |
| Stroke | 1. Met the diagnostic criteria by Chinese Stroke Association guidelines 2023 updates^1^ |
| Dementia | 1. Previously diagnosed with dementia 2. Met the national diagnostic criteria for dementia in the 2024 updated NIA-AA guidelines^2^ or Chinese national guidelines^3,4^ |
| Cognitive decline | Met the Chinese national diagnostic criteria mild cognitive impairment (2018) ^5^ |
| Primary Headache | Migraine, tension-type headache, and cluster headache followed the Chinese guidelines^6^ and the International Classification of Headache Disorders, 3rd Edition^7^. |
| Intracranial Infections | 1. Etiological diagnoses in cases of suspected intracranial infections were primarily based on cerebrospinal fluid (CSF) analysis. 2. When CSF data were unavailable, diagnoses were supported by clinical history, treatment response, and supplementary investigations. |

Reference

1. Liu, L. *et al.* Chinese Stroke Association guidelines for clinical management of ischaemic cerebrovascular diseases: executive summary and 2023 update. *Stroke Vasc. Neurol.* **8**, e3 (2023).

2. Jack, C. R. *et al.* Revised criteria for diagnosis and staging of Alzheimer’s disease: Alzheimer’s Association Workgroup. *Alzheimers Dement. J. Alzheimers Assoc.* **20**, 5143–5169 (2024).

3. National Center for Neurological Disorders, Xuanwu Hospital, Capital Medical University, National Center for Chronic and Noncommunicable Disease Control and Prevention, Chinese Center for Disease Control and Prevention, National Health Commission Capacity Building and Continuing Education Center, China Population and Development Research Center & Project Group of "Blue Paper on Alzheimer′s Disease in China ". *Blue paper on Alzheimer′s disease in China(simplified version)*. *Chinese Medical journal* 2701–2727 (Chinese Medical journal, 2024).

4. Guidelines Writing Group for Dementia and Cognitive Impairment in China. 2018 Chinese Guidelines for the Diagnosis and Treatment of Dementia and Cognitive Impairment (Part I): Diagnostic Criteria for Dementia and its Classification. *Natl. Med. J. China* 965–970 (2018).

5. Writing Group of Chinese Guidelines for Diagnosis and Treatment of Dementia and Cognitive Impairment, Professional Committee of Cognitive Impairment Diseases, Neurologist Branch of Chinese Medical Doctor Association. Chinese guidelines for diagnosis and treatment of dementia and cognitive impairment (V): diagnosis and treatment of mild cognitive impairment. *Natl. Med. J. China* **98**, 1294-1301.V (2018).

6. Wang, Y. Chinese evidence, practice guidelines and criteria for the diagnosis and treatment of primary headache. *Chin J Neurol* **56**, 587–590 (2023).

7. Headache Classification Committee of the International Headache Society (IHS) The International Classification of Headache Disorders, 3rd edition. *Cephalalgia Int. J. Headache* **38**, 1–211 (2018).
